# Combined immunodeficiency caused by pathogenic variants in the *ZAP70* C-terminal SH2 domain

**DOI:** 10.3389/fimmu.2023.1155883

**Published:** 2023-05-29

**Authors:** Cédric Mongellaz, Rita Vicente, Lenora M. Noroski, Nelly Noraz, Valérie Courgnaud, Javier Chinen, Emilia Faria, Valérie S. Zimmermann, Naomi Taylor

**Affiliations:** ^1^ Institut de Génétique Moléculaire de Montpellier, University of Montpellier, Centre National de la Recherche Scientifique (CNRS), Montpellier, France; ^2^ Immunology, Allergy and Rheumatology Section, Department of Pediatrics, Texas Children’s Hospital, Baylor College of Medicine, Houston, TX, United States; ^3^ Immunoallergy Department, Coimbra Hospital and University Centre (CHUC), Coimbra, Portugal; ^4^ Pediatric Oncology Branch, Center for Cancer Research, National Cancer Institute, National Institutes of Health (NIH), Bethesda, MD, United States

**Keywords:** primary immunodeficiencies (PID), ZAP-70, Syk, TCR zeta chain, SH2 mutations, autoimmunity, phosphate binding pocket, inborn errors of immunity (IEI)

## Abstract

**Introduction:**

ZAP-70, a protein tyrosine kinase recruited to the T cell receptor (TCR), initiates a TCR signaling cascade upon antigen stimulation. Mutations in the *ZAP70* gene cause a combined immunodeficiency characterized by low or absent CD8+ T cells and nonfunctional CD4+ T cells. Most deleterious missense *ZAP70* mutations in patients are located in the kinase domain but the impact of mutations in the SH2 domains, regulating ZAP-70 recruitment to the TCR, are not well understood.

**Methods:**

Genetic analyses were performed on four patients with CD8 lymphopenia and a high resolution melting screening for *ZAP70* mutations was developed. The impact of SH2 domain mutations was evaluated by biochemical and functional analyses as well as by protein modeling.

**Results and discussion:**

Genetic characterization of an infant who presented with pneumocystis pneumonia, mycobacterial infection, and an absence of CD8 T cells revealed a novel homozygous mutation in the C-terminal SH2 domain (SH2-C) of the *ZAP70* gene (c.C343T, p.R170C). A distantly related second patient was found to be compound heterozygous for the R170C variant and a 13bp deletion in the *ZAP70* kinase domain. While the R170C mutant was highly expressed, there was an absence of TCR-induced proliferation, associated with significantly attenuated TCR-induced ZAP-70 phosphorylation and a lack of binding of ZAP-70 to TCR-ζ. Moreover, a homozygous ZAP-70 R192W variant was identified in 2 siblings with combined immunodeficiency and CD8 lymphopenia, confirming the pathogenicity of this mutation. Structural modeling of this region revealed the critical nature of the arginines at positions 170 and 192, in concert with R190, forming a binding pocket for the phosphorylated TCR-ζ chain. Deleterious mutations in the SH2-C domain result in attenuated ZAP-70 function and clinical manifestations of immunodeficiency.

## Introduction

Inborn errors of immunity (IEI), also known as primary immunodeficiencies (PID), comprise a heterogeneous group of disorders, generally resulting from genetic mutations that negatively impact immune cell development and function. Within this group of diseases, severe combined immunodeficiencies (SCID) are characterized by defective T and B lymphocyte differentiation that are almost universally fatal in infancy. In these patients, replacement of the mutant hematopoietic stem cells (HSCs), either by HSC transplantation or by genetic correction of these progenitors, represents a critical therapeutic approach (1). While there has been a massive improvement in the outcome of PID patients following transplantations, significant short-term and long-term complications are still reported (2).

The broad spectrum of pathological features of IEIs, even in the context of a single affected gene, has made it difficult to define an optimal transplant protocol. Nevertheless, genetic diagnosis has been found to be associated with improved outcome of SCID patients undergoing HSCT (3, 4) and newborn screening, based on low or absent T cell receptor excision circles (TRECs), a biomarker for thymic output of newly differentiated T cells has accelerated the diagnosis and treatment of affected infants (5). Importantly though, not all IEI are associated with decreases in TRECs that fall below the threshold, hampering the diagnosis of this subset of immunodeficiencies. One such example is the immunodeficiency due to mutations in the *ZAP70* protein tyrosine kinase (PTK) gene, at least during the neonatal period (6, 7).

**Figure 1:**
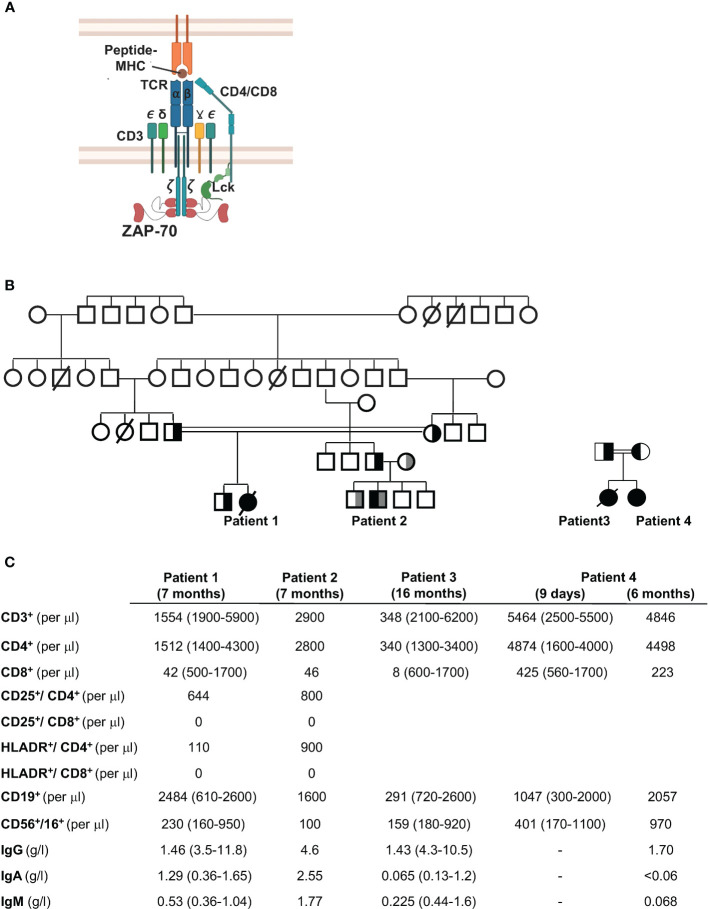
Family trees and immunological phenotypes of four patients with CD8 lymphopenia. **(A)** Schematic representation of the T cell receptor (TCR) showing the CD3 chains and recruitment of ZAP-70 to the Lck-phosphorylated immunoreceptor tyrosine–based activation motifs (ITAMs) in the TCR*_ζ_* chain. **(B)** Pedigree of the four patients. Patient 1 was the offspring of a consanguineous marriage between first cousins (double line). The father of Patient 2 was from the same fraternity as Patient 1 (first cousins) while the mother was unrelated. Patients 3 and 4 are siblings, offspring of a consanguineous marriage. **(C)** Immunological features of the four patients are shown at 7 months of age (Patients 1 and 2), 16 months (Patient 3), and 9 days and 6 months (Patient 4). All values are presented relative to age-matched reference values (presented in parentheses). Patients received IVIg.

ZAP-70 is recruited to the T cell receptor (TCR) following Lck-induced phosphorylation of the immunoreceptor tyrosine–based activation motifs (ITAMs) in the TCR*_ζ_* chain (8, 9). Binding of ZAP-70 to the TCR, via its two SH2 domains (N- and C-terminal), results in conformational changes; full activation of ZAP-70 then occurs through both Lck-induced phosphorylation and autophosphorylation (10–12) (**Figure 1A**). ZAP-70 is recruited into a signaling cluster at the membrane (13) with amplification of the cascade mediated through a cycle of ZAP-70 recruitment, activation, and release (14). The phosphorylation of downstream ZAP-70 effectors such as LAT and SLP-76, mediated at least in part through ZAP-70 S-acylation (15), propagates downstream TCR signaling (16, 17). In the absence of ZAP-70 recruitment, there is an asymmetric block in T cell differentiation in the human thymus, with the development of CD4^+^ but not CD8^+^ T cells. However, the peripheral CD4^+^ T cells in these patients are not functional, due to abnormal TCR signaling (18–20). Interestingly though, mice with ZAP-70 deficiency exhibit an earlier block in T cell differentiation in the thymus with a complete absence of peripheral T cells, likely due to decreased compensation by the related Syk PTK during the developmental process (21–29). Mutations in the *ZAP70* gene can be characterized in three groups: 1) the majority of patients exhibit a complete loss of expression and/or function of the ZAP-70 protein; 2) a smaller group present with hypomorphic mutations; and 3) a single patient with compound heterozygous mutations resulting in both loss-and gain-of-function mutations has been reported (30–35). While many of the mutations resulting in a complete loss of ZAP-70 function have targeted the kinase domain, the impact of SH2 mutations on ZAP-70 function are only incompletely understood. Here, we present 4 patients with two distinct mutations in the C-terminal SH2 domain, R170C and R192W, the development of a rapid genotyping assay by high resolution melting curve analysis, and the impact of these mutations on binding to the phosphate moiety of the phosphorylated TCR-ζ chain ITAMs.

## Results

### Patients

Third degree cousins, the product of consanguineous and non-related parents, respectively (**Figure 1B**), presented with a range of ailments during infancy. The first patient presented at 6 months of age to the Coimbra University Hospital, Portugal, with *Pneumocystis carinii* pneumonia, tuberculoid granulomas, and failure to thrive. She was started on IVIG and trimethoprim/sulphamethoxazole prophylaxis but did not receive a HSCT at 7 months of age because of the absence of an HLA-identical donor and after that time, the parents refused transplant. Despite recurrent *Pneumocystic jiroveci* pneumonia at 7, 25, and 28 months of age, the patient did relatively well without severe bacterial or viral infections (until 5.5 years of age). She did though present with autoimmune symptoms including alopecia areata as well as papules and nodules predominantly on the face and limbs. She died from infectious complications at 7 years of age.

The second patient presented at 6 months of age with *Pneumocystis jiroveci* pneumonia and mycobacterial infections with subsequent recurrent respiratory and gastrointestinal infections. He also had a left axillar adenitis. He was successfully transplanted at 15 months of age with cryopreserved umbilical cord stem cells from a healthy HLA-identical brother.

Peripheral blood analyses from both patients at 7 months of age showed a normal number of B cells, with low or normal NK cell numbers, and immunoglobulin levels (following IVIg supplementation). Lymphocyte immunophenotyping revealed decreased T lymphocyte numbers in Patient 1 but not Patient 2 with normal levels of CD4^+^ T helper cells in both patients. Notably though, both patients presented with extremely low levels of CD8^+^ T lymphocytes relative to controls (**Figure 1C**).

A third female patient, the product of consanguineous parents, presented at Texas Children’s Hospital with fever and respiratory distress at 16 months of age. Her symptoms had started four months after receiving her first dose of the MMR vaccine. She had a maculopapular rash on her face and extremities with an unusual clustering of the rash, palatal ulcers, and autoimmunity manifested as bilateral hand arthritis, with inflammation at the proximal, intermediate and distal interphalangeal joints of both hands. She had progressive deterioration of her lung function and expired. Microbiology and pathology studies demonstrated pneumonitis and hepatitis induced by the measles vaccine strain, and ruled out other pathogens. Immunologic evaluation uncovered a severe T-cell lymphopenia with nearly absent CD8 T cells (**Figure 1C**). Lymphocyte proliferation to mitogens were decreased and titers to diphtheria, tetanus, and pneumococcal serotypes were absent.

The fourth patient, a second girl, was born to same parents as Patient 3. Prenatal genetic diagnosis was declined. Lymphocyte phenotype at nine days of age, as well as at 6 months of age showed decreased CD8 T cells (**Figure 1C**). She received an umbilical cord transplant from a full matched donor at 6 months of age, resulting in a successful immune reconstitution.

### Identification of *ZAP70* mutations in the C-terminal SH2 domain in four patients

**Figure 2:**
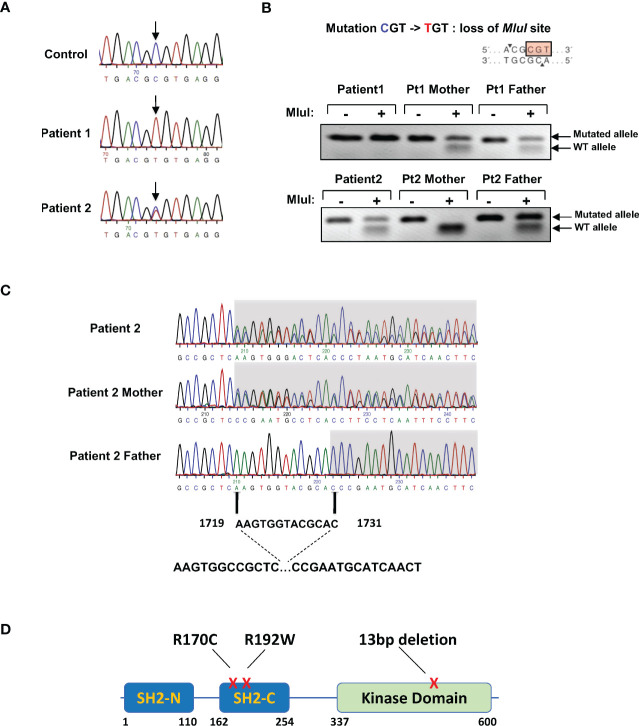
Presence of ZAP-70 SH2-C domain mutations in the four patients. **(A)** ZAP-70 specific products were amplified from the genomic DNAs of a healthy individual and the two patients. Sequencing over exon 4 harboring the C-terminal SH2 domain revealed a homozygous mutation in R170 in patient 1 and a heterozygous mutation in patient 2. The associated electropherograms showing the R170C mutation are presented. **(B)** Presentation of the R170 mutation showing a loss of the *MluI* restriction site (top). Digestion of DNAs from patient 1, patient 2, and both parents are presented, with the presence of the WT and mutated alleles indicated with arrows (n=3 for patient 1 and n=2 for patient 2). **(C)** Genomic DNA sequence analysis of the kinase domain of *ZAP70* in patient 2 and his parents, revealing a 13bp heterozygous deletion from bp1719-1731 in the patient and his mother. **(D)** Schematic presentation of the *ZAP70* gene showing the N- and C-terminal SH2 domains (SH2-N and SH2-C) as well as the kinase domain. The positions of the mutations detected in patients 1, 2, 3, and 4 are indicated.

As the immunophenotype of the two patients is characteristic of ZAP-70 immunodeficiency (11, 19, 22, 36, 37), the *ZAP-70* gene was sequenced. Molecular analyses of the first patient revealed a homozygous C to T mutation in exon 4 (bp 266), resulting in an Arg to Cys mutation at position 170 of the C-terminal SH2 domain (SH2-C, **Figure 2A**). Her parents were both heterozygous carriers for this mutation, as confirmed by the loss of an *MluI* restriction site at this position (**Figure 2B**). The father of patient 2 was the first degree cousin of both the mother and father of patient 1. The parents of this child were not related and the patient carried the R170C mutation in the SH2-C from his father and a second heterozygous 13bp deletion (nucleotides 1719-1731) in exon 12 of the kinase domain (AAGTGGTACGCAC, **Figure 2C**). This latter mutation has previously been shown to result in an absence of ZAP-70 expression (20, 33, 38). Genetic analysis of patients 3 and 4 (Correlagen Inc, MA) revealed a homozygous variant (p.R192W) in the SH2 domain of the *ZAP70* gene and a schematic representation of these mutations is shown in **Figure 2D**. Of note, > 18 mutations in the *ZAP70* kinase domain have been reported with only 3 mutations found in the C-terminal SH2 domain (33).

### Rapid diagnosis of ZAP-70 mutations in the C-terminal SH2 and kinase domains

**Figure 3:**
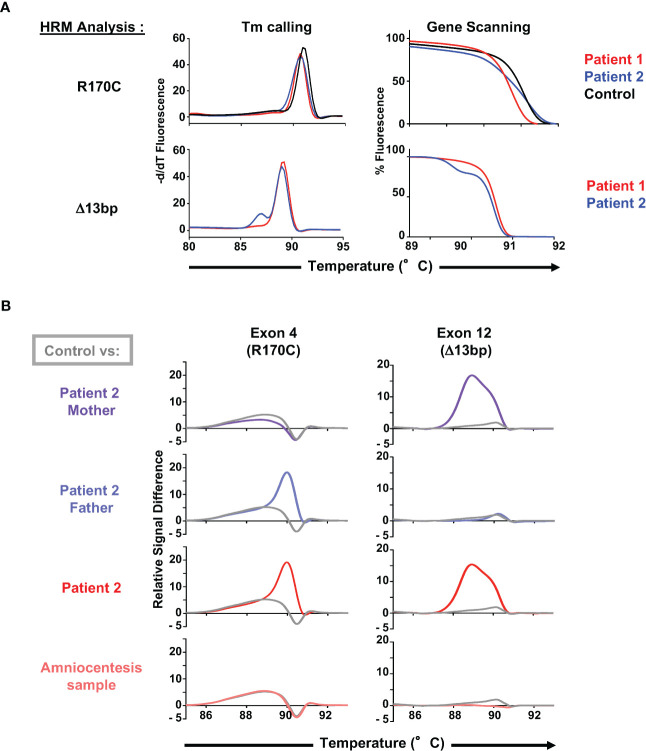
Use of HRM dyes for an optimal and rapid diagnosis of the *ZAP70* mutations. **(A)** Melting curve analysis (Tm calling, left panel) and gene scanning analysis (normalized melting curve representation, right panel) of the high resolution melting (HRM) PCR-amplified 163bp genomic product including the C>T mutation (R170C) in the *ZAP70* gene (patients 1 and 2, n=2) as well as the 139bp product including the 13bp deletion (patient 2, n=2). **(B)** Evaluation of the C>T mutation (R170C) and the 13bp deletion using HRM combined with a difference plot representation of the gene scanning analysis. Profiles of both parents of patient 2, patient 2, and an amniocentesis sample from family 2 are presented (n=2), with each profile compared to a healthy control (presented in grey).

While direct DNA sequencing is the gold standard for the evaluation of genetic mutations, these analyses are costly and often take time. Furthermore, they are not readily available in areas with limited access and resources. This issue is also especially difficult in the context of neonatal diagnosis, as was the case for the family of patient 2. The high-resolution melting (HRM) technique has been used to scan for mutations across several important genes. Here, we evaluated the detection of the single C>T mutation (R170C) and the 13bp deletion using primers designed to amplify a 163bp and 139bp qPCR products, respectively. Initial analyses of the R170C melting curve by the Tm calling analysis were not able to distinguish patient and healthy donor curves but a normalization melting curve representation was able to discriminate the homozygous and heterozygous C>T mutation in patient 1 and 2, respectively (**Figure 3A**, top). While the 13bp deletion could be distinguished from the wt exon 12 by Tm calling analysis, both mutations were easily distinguished by difference plot representations of the HRM screening in patient 2 and his parents (**Figure 3B**). This analysis was therefore used to evaluate an amniocentesis sample from the mother of patient 2 and importantly, results from the amniocentesis sample were available in <6 hours, revealing the presence of two WT alleles (**Figure 3B**). Testing of this single gene disorder was performed by sequencing of the *ZAP70* gene but it is important to note that this required 2 weeks. Thus, this methodology was able to successfully discriminate a complex genotype and allowed for rapid diagnosis from an amniocentesis sample.

### Abrogated TCR-mediated proliferation of patient T cells harboring the R170C mutation despite high ZAP-70 expression

**Figure 4:**
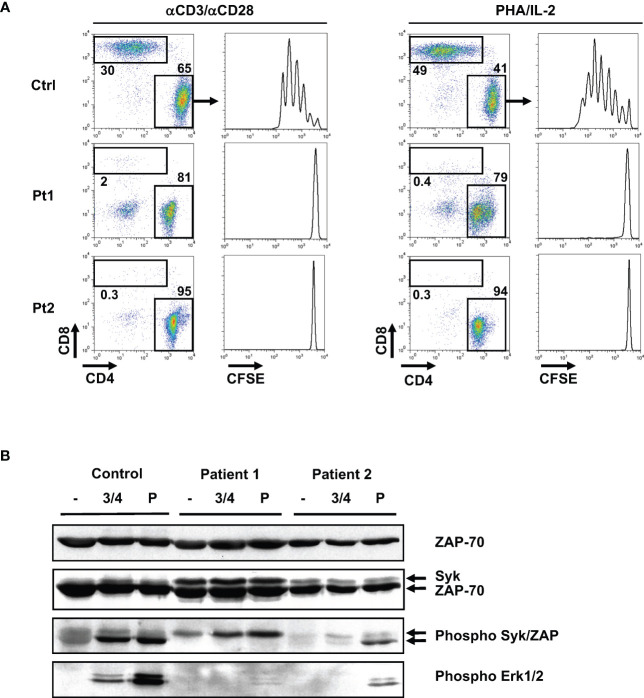
Absence of TCR-induced proliferation in patient T cells is associated with defective phosphorylation of the ZAP-70 protein tyrosine kinase. **(A)** T cells from the two patients and a healthy control were labeled with CFSE and activated with anti-CD3/anti-CD28 antibodies or with PHA/IL-2. Following 5 days of stimulation, the relative percentages of CD4 and CD8 T cells were monitored and representative dot plots are shown. The CFSE proliferation profiles of gated CD4 T cells are presented. **(B)** Control and patient T cells were activated by anti-CD3/anti-CD4 mAb crosslinking (3 min) or with pervanadate (5 min). Total ZAP-70 and Syk expression were monitored by immunoblotting with a monoclonal ZAP-70 specific antibody and a polyclonal antibody recognizing Syk, respectively. Phosphorylation of Syk and ZAP-70 were assessed using an anti-phospho-Syk/ZAP-70 antibody (Y319/Y352) and the respective phosphorylated bands are indicated with arrows (middle panel). Phosphorylation of Erk1/Erk2 was monitored with a pan-phospho-MAPK antibody (lower panel; n=2 for patient 1 and n=1 for patient 2).

The impact of the R170C mutation was first evaluated at the level of T cell proliferation. As the patients had almost no CD8 T cells, T cell receptor (TCR)-stimulated proliferation of the CD4 T cell subset in patients 1 and 2 was compared to CD4 T cells from a healthy control. T cells were labeled with the CFSE proliferation dye and stimulation of control CD4 T cells with either anti-CD3/anti-CD28 mAbs or PHA with IL-2 resulted in at least 5 divisions. In contrast, there was a complete absence of proliferation of patient CD4 T cells in response to CD3/CD28 or PHA/IL-2 (**Figure 4A**).

Importantly, levels of the R170C ZAP-70 protein in patient T cells were equivalent to wt ZAP-70 detected in healthy controls (**Figure 4B**, top panel). We also assessed expression of the ZAP-70-related Syk protein as high expression of the latter has been proposed to lead to an abnormal activation and differentiation of CD4+ T lymphocytes in ZAP-70-deficient patients (26, 39). Interestingly, Syk was detected in both patients, albeit at higher levels in patient 1 where Syk was also phosphorylated (**Figure 4B**, middle panel). Notably though, the absence of ZAP-70 phosphorylation in TCR-stimulated patient T cells resulted in aberrant downstream proximal TCR signaling with the lack of phosphorylation of the ERK1/ERK2 MAPK. This was specifically due to defective TCR signaling as activation of patient 2 T cells with pervanadate, an agent that activates intracellular kinases independently of the TCR, resulted in phosphorylation of both ZAP-70 and ERK1/ERK2. Together, these data show that the R170C mutation in ZAP-70, even with high levels of Syk, adversely impacts the proximal TCR signaling cascade in patient T cells.

### Expression of the R170C ZAP-70 mutant in a ZAP-70^-/-^/Syk^-/-^ T cell line abrogates proximal TCR signaling and association with the TCR ζ-chain

**Figure 5:**
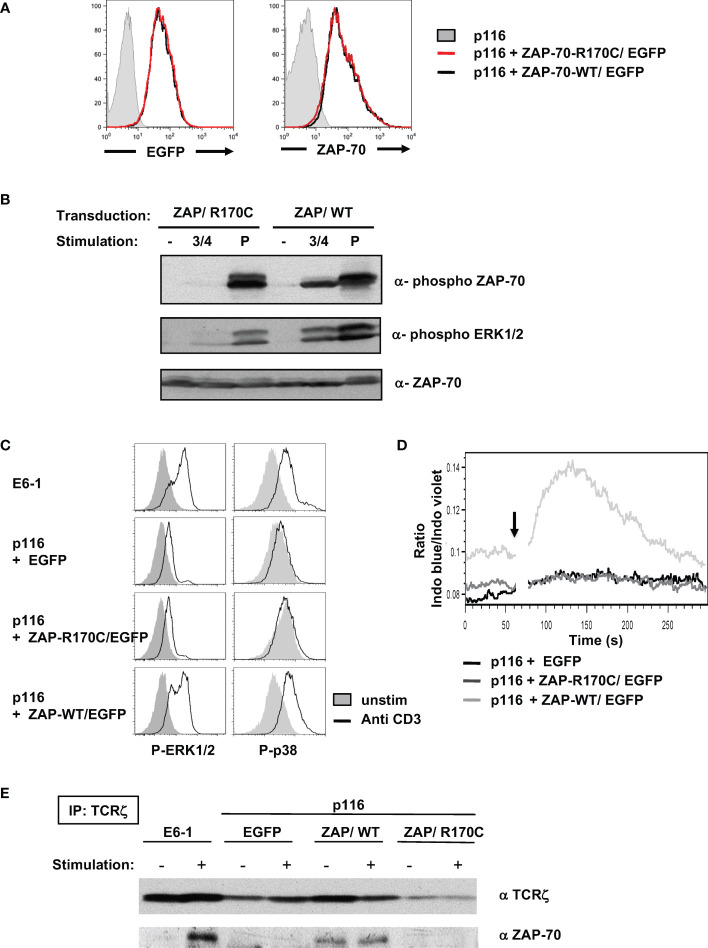
Ectopic expression of the R170C mutant ZAP-70 does not reconstitute proximal TCR signaling in a ZAP-70-deficient Jurkat T cell line. **(A)** The ZAP-70^-/-^ p116 Jurkat cell line was transduced with retroviral vectors harboring EGFP together with the ZAP-70 R170C mutant (ZAP/R170C) or WT ZAP-70 (ZAP/WT). EGFP+ cells were sorted and the level of EGFP expression in the purified populations is shown compared to the parental p116 ZAP-70^-/-^ Jurkat cells (left filled histogram). The levels of ZAP-70 in the different Jurkat cells were monitored by intracellular staining with a monoclonal anti-ZAP-70 antibody (right histograms). **(B)** The potential for T cell stimulation, via CD3/CD4 crosslinking or pervanadate, to induce phosphorylation of ZAP-70 and ERK1/ERK2 MAPK was monitored after a 5 minute stimulation using the appropriate anti-phospho antibodies. Representative immunoblots together with total ZAP-70 levels are shown. **(C)** ERK1/2 and p38 phosphorylation were monitored by intracellular staining in control Jurkat cells (E6.1, ZAP-70^+/+^), as well as in EGFP, ZAP-R170C/EGFP, and ZAP-WT/EGFP-transduced p116 cells. Representative histograms showing ERK1/2 and p38 phosphorylation in non-stimulated (filled histograms) as compared to TCR-stimulated cells (anti-CD3 mAb, open histograms) are presented. **(D)** Calcium flux was monitored by flow cytometry following loading with Indo-I and activation with an anti-CD3 mAb (arrow). Intracellular calcium levels are presented in arbitrary units as a function of time (seconds). **(E)** TCRζ was immunoprecipitated from control ZAP-70^+/+^ Jurkat cells (E6.1) as well as from EGFP-, ZAP-R170C/EGFP-, and ZAP-WT/EGFP-transduced ZAP-70^-/-^ p116 cells after a 3 minute anti-CD3 mAb stimulation. Representative immunoblots showing immunoprecipitated TCRζ levels as well as ζ-associated ZAP-70 are presented (n=2 for each panel).

To specifically evaluate the impact of the R170C mutation in ZAP-70, this mutant was stably introduced into the p116 ZAP-70^-/-^/Syk^-/-^ Jurkat T cell line which has been shown to support TCR signaling following introduction of ectopic ZAP-70 (40). P116 cells were transduced with lentiviral vector harboring either WT or R170C ZAP-70 together with the EGFP reporter gene. Transduced cells were sorted on the basis of EGFP expression and intracellular staining revealed similar levels of WT and R170C mutant ZAP-70 (**Figure 5A**). Following TCR stimulation via anti-CD3/anti-CD4 mAb crosslinking, only WT ZAP-70 was phosphorylated, despite similar levels of ZAP-70 protein, and ZAP-70 phosphorylation was associated with phosphorylation of ERK1/ERK2 (**Figure 5B**). Notably though, pervanadate stimulation resulted in high levels of ERK1/ERK2 phosphorylation irrespective of the ZAP-70 mutation, showing that proximal signaling was maintained when ZAP-70 activation was circumvented (**Figure 5B**). Furthermore, anti-CD3-mediated phosphorylation of ERK1/2 as well as p38 MAPK, as assessed by intracellular staining, was induced to similar levels in ZAP-70-reconstituted p116 cell as compared to parental ZAP-70+/+ Jurkats (E6-1) while phosphorylation of these downstream substrates was not detected in either the EGFP-transduced or R170C ZAP-70-transduced p116 cells (**Figure 5C**). These data correlated with the mobilization of intracellular calcium, another proximal signaling event (**Figure 5D**). These data reveal the defective nature of early TCR-mediated signaling events in T cells expressing the R170C ZAP-70 mutant.

Previous studies have revealed the importance of the R192 residue in the function of ZAP-70 as an R192W mutation, also in the SH2-C domain, results in impaired binding of the mutant ZAP-70 to the phosphorylated ζ-chain (41, 42). To assess the potential of the R170C ZAP-70 to bind to TCR-ζ, we performed TCR-ζ immunoprecipitations on parental Jurkat (E6-1) as well as the ZAP-70^-/-^/Syk^-/-^ p116 cells reconstituted with EGFP, WT ZAP-70, and R170C ZAP-70. As shown in **Figure 5E**, ZAP-70 only co-immunoprecipitated with TCR-ζ in cells harboring a WT ZAP-70. Thus, these data highlight the importance of the R170 residue in mediating association with the TCR-ζ chain.

### Critical role of SH2-C domain arginine residues in ZAP-70 function and structure

**Figure 6:**
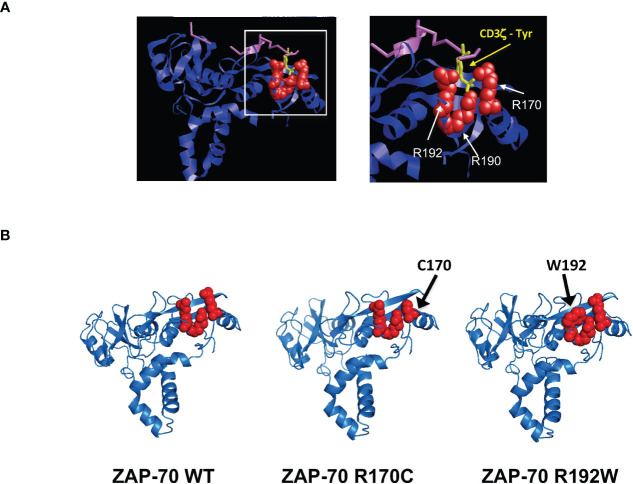
Pathological arginine mutations in the ZAP-70 C-terminal SH2 domain interact with the TCR-ζ-ITAM binding pocket. **(A)** Ribbon representation of the 3-dimensional structure of human ZAP-70 highlighting the interaction with a 19-mer TCR-ζ peptide containing phosphotyrosine 304. The binding of ZAP-70 to the phosphate within TCR-ζ (purple) occurs via hydrogen binding to three residues; arginine 170, 190 and 192 and the positions of these amino acids relative to TCR-ζ are presented (pdb entry 2OQ1). **(B)** Alphafold2-predicted structures of the ZAP-70 SH2 domains are presented for WT ZAP-70 as well as the R170C and R192W mutants. Amino acids in the TCR-ζ binding pocket are shown in red.

As indicated above, Arg-192 has been detected as a compound heterozygous mutation, in a patient who also presented with a kinase domain mutation (R360P) that weakens the autoinhibitory conformation of ZAP-70 (41, 42). Indeed, the R360P mutation in conjunction with the R192 mutation was associated with autoimmune manifestations in this patient (41, 42). It was therefore of much interest to better understand the impact of an R192 mutation alone, in the absence of a gain of function ZAP-70 mutation. The clinical course of Patients 3 and 4 demonstrate the pathogenicity of this mutation. The critical nature of mutations in Arg-170 and Arg-192 of ZAP-70 shown here – together with previous data showing the importance of Arg-190 and Arg-192 of SH2-C in interacting with the phosphate moiety of the phosphorylated TCR-ζ chain ITAM (43–45) – led us to evaluate the potential structure in the phospho-tyrosine binding pocket. Indeed, recent work highlights a network of non-covalent interactions that result in the coupling of the two SH2 domains to the doubly-phosphorylated ITAMs (46). Using PyMOL (47) to generate a structural representation of the ZAP-70 SH2 domains associated with the TCR-ζ chain, the prominent positioning of these three arginine is highlighted (**Figure 6A**). Furthermore, using the AlphaFold2 protein structure database (48, 49), the predictions reveal changes in the structure of the residues associating with TCR-ζ upon mutation of arg-170 to cys and arg-192 to trp (**Figure 6B**). These results strongly suggest that the pathological consequences of mutations in R170 and R192 of ZAP-70 are due to changes in the binding of ZAP-70 to TCR-ζ.

## Discussion

We report a novel mutation in the C-terminal SH2 domain of ZAP-70, resulting in a homozygous arginine-170 to cysteine mutation and compound heterozygosity with a previously described 13bp deletion leading to a translational frameshift after residue 503 (K504_P508delfsX35) (20). A second arginine mutation in the SH2-C domain has recently been described, but the affected child was found to be compound heterozygous for a R360P mutation (42). As the R360P mutation enhanced signaling due to a reduction in the autoinhibitory conformation of ZAP-70 (41), it was of interest to determine the effect of a homozygous R192W mutation on the function of ZAP-70. Notably, both R170C and R192W mutations negatively impacted the binding of ZAP-70 to the TCR ζ-chain, resulting in a complete absence in TCR-induced proliferation (this study and (42)) and all patients presented with immunodeficiency and autoimmunity, in the absence of activating mutations.

Implementation of newborn screening for SCID, evaluated as a function of low or absent TRECs on dried blood spots, promotes prompt diagnosis and treatment (5, 50–52). While infants with IEI due to mutations in *ZAP70* might not present with TREC numbers that are below the threshold for diagnosis (6, 7), it is important to note that the vast majority of these patients die from infections if they are not treated by HSCT. The two ZAP-70-deficient patients who received a HSCT are alive and well with full immune reconstitution. Unfortunately, the 2 non-transplanted patients died of infectious complications, highlighting the need for rapid diagnosis. Schroeder et al. developed a PCR-based diagnostic assay for the c.1624-11G>A mutation in *ZAP70*, identified as the causative mutation in all affected Mennonite probands (53). This type of analysis will advance early interventions. Here, we developed a simple and rapid HRM-based assay which allowed the *ZAP70* genotypes in the affected family to be evaluated within 6 hours of sample arrival. Extending these types of assays, especially to environments where genomic sequencing is not readily available, will improve carrier identification, diagnosis, and treatment.

The vast majority of *ZAP70* mutations that are responsible for a pathological immunodeficiency result in a dramatically reduced TCR-induced signaling *in vitro*. As such, it remains unclear as to how these mutations lead to autoimmunity in patients. In the case of the R170C and R192 mutations, the autoimmune phenotypes of the patients highlight the potential for defective T cells to respond to *in vivo* stimulatory signals. Interestingly, ZAP-70-independent stimulation of MAPK proteins has been reported following CD2 crosslinking (54) and activated T cells have been detected in ZAP-70-deficient patients (39). These data suggest that other PTKs may promote pathological T cell responses. Although the ZAP-70-related Syk PTK is downregulated during thymocyte differentiation under physiological conditions (55), this kinase has been detected in mature CD4+ T cells from ZAP-70-deficient patients, altering T cell activation (26, 29). High Syk levels were also detected in the patient with the R170C homozygous mutation reported here, potentially accounting for their low level of severe infections during a 3-year period. Nevertheless, other potential compensatory mechanisms may have contributed to the patient’s wellbeing. While a rate-limiting threshold promoting ZAP-70 responses to TCR signaling has been reported in mice (56), ZAP-70-deficient patients can present without severe infections during the first year of life despite undetectable ZAP-70 expression (30, 57). Thus, the crosstalk between proximal T cell signaling molecules is clearly complex. Indeed, T cells with mutations in the adapter protein linker for activation of T cells (LAT), relaying T cell antigen receptor triggering to downstream T cell responses, are able to maintain nuclear factor (NF) κB signaling even though ERK signaling is abrogated (58). The finding that LAT-deficient patients, like many ZAP-70-deficient patients, present with immunodeficiency as well as autoimmunity raises questions concerning the intricate crosstalk that regulate T cell responses from TCR signaling hubs (58–60).

The results presented here reveal the pathogenicity of variants in the *ZAP70* SH2 domain. Resolution of the crystal structure of ZAP-70 revealed connections between the two SH2 domains, producing an interface that results in binding to the doubly-phosphorylated ITAM of the TCR ζ-chain (44). Recent work has further shown that the two SH2 domains are allosterically coupled via noncovalent interactions upon ITAM binding (46) and an R192A mutant exhibits only minimal affinity for the ITAM (61). In conjunction with data showing a role for ZAP-70 as a structural protein regulating integrin-mediated control of actin (62), our results highlight the critical role of the R170 and R192 residues in ZAP-70 folding in association with the TCR ζ-chain. All together, we find that these mutations are pathogenic; defective folding of R170C and R192W ZAP-70 variants results in clinical manifestations of immunodeficiency and autoimmunity.

## Materials and methods

### T cell isolation and cell culture

Blood samples were harvested from patients and their related family members at the hematological department of Coimbra’s Hospital, Portugal (families harboring the R170C mutation) or at Texas Children’s Hospital, USA (family harboring the R192W mutation). Samples were obtained after signed informed consent and approval by the IRBs of the two hospitals. T cells were purified by centrifugation on a Ficoll gradient (Ficoll Histopaque 1077, Merck) following incubation with the RosetteSep T Cell Enrichment kit (StemCells Technologies) according to the manufacturer’s instructions. Primary T cells were cultured in RPMI 1640 medium supplemented with 10%FCS and 1% Penicillin-Streptomycin for functional analysis.

The E6-1 (TIB-152, ATCC) and ZAP-70^-/-^/Syk^-/-^ p116 (40) Jurkat cell lines were cultured in RPMI 1640 medium supplemented with 10% FCS and 1% penicillin-streptomycin.

### Genetic analyses and Sanger sequencing

T cells (10e6) were lysed and RNA were extracted using the RNA Easy mini kit (Qiagen) according to the manufacturer’s instructions. cDNA was obtained by elongation using oligo-dT primers with the M-MuLV Reverse Transcriptase Reaction kit (Qiagen) and PCR-amplified exons were sequenced by Sanger’s Method.

### Allele specific restriction analysis

Genomic DNA (10ng), extracted with the QiaAMP DNA Blood Mini kit (Qiagen), was amplified using the Sybr Green I Master Mix (Roche) with exon 4 primers specific for the R170C mutation (Forward: CTGGAGCAGGCCATCATCAGC; Reverse: GCCCCACATACAGGAACTTG) using the following program; 5 sec 95°C, 10 sec 60°C, 10 sec 72°C, for 30 cycles. Products from the R170C qPCR were purified using the Nucleospin Gel PCR cleanup kit (Macherey-Nagel) and digested with the *MluI* restriction enzyme (1U) for 30min at 37°C to assess the mutation-induced disruption of this restriction enzyme recognition site. *MluI*-digested DNA fragments were separated on a 1.8% agarose gel and the image was captured using an UV-illuminator (Ingenius SynGene).

### High resolution melting analysis by qPCR

Genomic DNA was extracted as described above. Detection of the R170C mutation and the 13bp deletion was performed by Real-Time qPCR using the LightCycler 480 High Resolution Melting Master mix (Roche) on the LightCycler480 machine (Roche). Briefly, genomic extract (10ng) was amplified with exon 4 primers specific for the R170C mutation (Forward: CTGGAGCAGGCCATCATCAGC; Reverse: GCCCCACATACAGGAACTTG) and exon 12 primers specific for the 13bp deletion (Forward: GATCCAGCAGCATCTCCC; Reverse: CCTCCCACATGGTGACC) using the following program; 5 sec 95°C, 10 sec 60°C, 10 sec 72°C, for 30 cycles. An extensive HRM curve (from 62 to 95°C, rate 0.02°C/sec) was analyzed using LightCycler 480 II software.

### Flow cytometry analyses

To detect cell surface markers, cells were incubated with the appropriate fluorochrome-conjugated mAbs and expression was monitored in comparison with isotype controls. Antibodies against CD3, CD4, CD8, CD25, HLA-DR, CD19, CD20, and CD56 were from Beckman Coulter. Intracellular ZAP-70 was detected using PE-conjugated anti ZAP-70 (clone 1E7.2, eBioscience). T^202^Y^204^-phosphorylated ERK1/2 (Clone 20A, BD Biosciences), and T^180^/Y^182^-phosphorylated p38 (Clone 36/38, BD Biosciences) were detected after cell fixation and permeabilization (Cytofix, PhosFlow Buffer III, BD Biosciences).

For lymphocyte subset analysis, 100 µl of whole blood was incubated with mAbs against surface markers for 20 minutes in the dark at room temperature. Red cells were then lysed and washed before acquisition. For evaluation of TCR signaling, Jurkat cells transduced with the indicated vectors were starved overnight in RPMI media containing only 1% FCS. Cells (1x10^6^) were then activated in 100 µl RPMI with anti-CD3 (clone OKT3, 1 µg/ml) and anti-CD4 (clone 13B8.2, 1 µg/ml) mAbs for 1 min, and crosslinked by goat anti–mouse Fab’2 fragment (1:100) for 2 min. Cells were assessed on a FACS-CantoII or BD LSRII-Fortessa (BD Biosciences) and data were analyzed using Diva (BD Biosciences) or FlowJo (Tree Star) software.

### Proliferation assays

Proliferation was monitored as a function of CFSE dilution. CD3^+^ T cells were stained with 1μM CFDA-SE (Cell Trace CFSE kit, ThermoFisher) for 3 min and washed twice with PBS 2% FCS. T cells (1x10^6^) were subsequently plated into anti-CD3/anti-CD28 coated wells (clones OKT3 and 9.3 respectively, BioXCell) or stimulated with PHA (1μg/ml-Sigma) and rhIL2 (100IU/ml-Proleukin). Proliferation was evaluated at day 5 following staining with anti-CD4 APC (clone 13B8.2) and anti-CD8 PeCy7 (clone SFC121Thy2D3) mAbs and data acquired on a FACSCanto II Flow Cytometer (Becton Dickinson). Analyses were performed using FlowJo Software (v10–10.6.2).

### *ZAP70* mutagenesis

Site-directed R170C c>t mutagenesis was performed by amplification of the *BamHI*/*BstEII* fragment of the WT ZAP-70 cDNA from a previously reported lentiviral vector containing the EGFP reporter gene (63). Amplification was performed using the Expand Long Template kit (Expand High Fidelity template, Roche) with the following forward and reverse primers; cacagcagcctgacgtgtgaggaggccgagcg and cgctcggcctcctcacacgtcaggctgctgtg, respectively. The DNA template was digested with the *DpnI* restriction enzyme (1U) for 1h at 37°C. After sequencing, the *BamHI*/*BstEII* fragment harboring the R170C mutant was reinserted into the original pRRL PGK ZAP-70 vector and sequence verified.

### Generation of Jurkat cell lines expressing WT and R170C ZAP-70

Self-inactivating HIV-1-derived viruses were generated by transient co-transfection of 293T cells with the PsPax2 packaging vector, encoding Gag, Pol, Rev and Tat, a VSV-G envelope glycoprotein plasmid (pCMV-VSV-G), and the HIV-1 derived SIN vector encoding either the EGFP reporter gene alone, or WT ZAP-70 or the R170C ZAP-70 mutant with EGFP downstream of an IRES site (64). Viral supernatants were harvested 48 hours post-transfection, centrifuged, filtered, and used to transduce the ZAP-70^-/-^/Syk^-/-^ p116 cell line. Transduced p116 cells were sorted on the basis of EGFP expression on a BD FACSAria (BD Biosciences).

### Immunoblot analyses and immunoprecipitations

To assess expression of ZAP-70 and protein phosphorylation, T cells (1x10^6^) were stimulated with anti-CD3 (clone OKT3 (1 µg/ml) and anti-CD4 (clone 13B8.2 (1 µg/ml) mAbs for 1 min at 37°C, followed by a cross-linking with goat anti–mouse Ig (1:100) for 2 min. Cells were then lysed in 1% NP-40 detergent buffer (1% Nonidet P-40, 150 mM NaCl, 20 mM Tris, pH 7.4) containing 5μg/ml aprotinin (Sigma Aldrich), 1mM sodium orthovanadate (Sigma Aldrich) and 1mM sodium fluoride (Sigma Aldrich).

For immunoprecipitation of the TCR ζ chain in the different Jurkat clones, 1x10^7^ cells were activated as above, washed in ice-cold PBS, and cell pellets were lysed in 500µl of imuunoprecipitation (IP) Buffer (0.5% NP-40, 0.5 Sodium Deoxycholate, 150 mM NaCl, 20 mM Tris, pH8) supplemented with 1mM sodium orthovanadate. Lysates were incubated with an anti-TCR ζ antibody (2 μg, clone 6B10.2; Santa Cruz Biotechologies) for 1h at 4°C, followed by a 1h incubation with magnetic protein AG beads (Ademtech) at 4°C. Immunoprecipitated proteins were eluted and analyzed by immublotting as described below.

SDS-reduced samples were separated on a 4-20% polyacrylamide gel under SDS-denaturing conditions, and transferred electrophoretically to PVDF. Membranes were incubated overnight at 4°C with primary antibodies against ZAP-70 (clone 2F3.2), P-Tyr^319^ZAP-70/P-Tyr^352^Syk and P-T^202^Y^204^ ERK1/2 (CST), and then revealed by incubation with HRP-coupled secondary antibodies at room temperature for 1h (1:10.000 in PBS-Tween 1% milk). Immunoreactive bands were visualized by enhanced chemiluminescence (Pierce ECL Western, ThermoScientific).

### Calcium flux analyses

ZAP-70^-/-^/Syk^-/-^ p116 cells (2x10^6^) expressing EGFP, WT ZAP70, or R170C ZAP70 were loaded with 1µM of the ratiometric calcium indicator Indo-1 (Molecular Probes) for 30min at 37°C. Cells were then washed and analyzed; after 1 min of acquisition on a BD FACSAria (Becton Dickinson), cells were activated by addition of anti-CD3 (clone UCHT1) and anti-CD4 (clone 13B8.2) mAbs for 1 min followed by crosslinking using an anti-mouse Fab’2 fragment for 2 min. Calcium flux was analyzed as a function of the ratio of the green (filter BP 530/30nm) and violet emissions (filter BP 450/50nm) of Indo-1 using FlowJo software.

### Protein structure predictions

Predictions of the structure of the SH2 domains of ZAP-70, with or without the kinase domain (PDB 2OQ1), in association with the phosphorylated ITAMs of the TCR-ζ chain were determined using PyMOL, an open source molecular visualization system (47), as well as AlphaFold2, a leading computational method for predicting protein structure (48, 49).

## Data availability statement

The original contributions presented in the study are included in the article/supplementary material. Further inquiries can be directed to the corresponding authors.

## Ethics statement

The studies involving human participants were reviewed and approved by Coimbra Hospital and University Centre, Coimbra, Portugal; Baylor College of Medicine IRB, Texas. Written informed consent to participate in this study was provided by the participants’ legal guardian/next of kin.

## Author contributions

CM, RV, NN, EF, JC, VZ, and NT conceived the study. CM, RV, NN, VC, and VZ performed experiments. LN, JC, and EF provided patient care, collected samples and clinical data, and analyzed patient data. JC, VZ, and NT supervised the study. All authors participated in data analysis and discussions. CM, JC, VZ, and NT wrote the manuscript and all authors critically reviewed the manuscript and approved the final version.
